# Suppressing CDCA8/CDK1 improves oral squamous cell carcinoma by modulating proliferation, apoptosis, cell cycle and migration

**DOI:** 10.1007/s12672-025-03648-z

**Published:** 2025-10-15

**Authors:** Jiang Zhu, Chen Chu, Yu Xue, Wei Guan, Jianping Qiu, Aijun Guo, Weiming Chu

**Affiliations:** 1 Department of Stomatology, Affiliated Hospital of Nantong University, 226001 Nantong, China; 2 Department of Stomatology, Northern Jiangsu People’s Hospital, 225000 Yangzhou, China; 3 Department of Oral and Maxillofacial Surgery, Yangzhou Stomatology Hospital, 225000 Yangzhou, China

**Keywords:** Oral squamous cell carcinoma, CDCA8, CDK1, Cell cycle, Apoptosis

## Abstract

We aimed to clarify the role of CDCA8/CDK1 in the progression of oral squamous cell carcinoma (OSCC) and the potential mechanisms. To explore the CDCA8 expression in OSCC, clinical samples were collected. In vitro, CAL-27 cells were transfected with short hairpin RNA (shRNA) to silence CDCA8, and then proliferation, apoptosis, cell cycle and migration were evaluated. KEGG analysis was used to explore the causal association of CDCA8 and CDK1 during the development of OSCC. CDK1 inhibitor RO3306 and overexpression of CDK1 were used to clarify the regulatory role of CDK1 in OSCC. In vivo, mice were injected with shCDCA8 to deplete CDCA8 based on establishing a model of OSCC. We found that CDCA8 was highly expressed in OSCC tissues and cell lines. In vitro experiments, suppressing CDCA8 in CAL-27 cells improved OSCC, characterized by increased apoptosis and decreased proliferation and migration. Kyoto Encyclopedia of Genes and Genomes (KEGG) pathway enrichment analysis revealed that cell cycle formed a direct interaction with CDCA8 and inhibition of CDCA8 efficiently reduced levels of CDK1 and CDK2. Additional experiments showed that CDCA8-activated CDK1 was the key regulator of OSCC. In vivo, we verified that depletion of CDCA8 inhibited proliferation and metastasis of OSCC. CDCA8-activated CDK1 acts a crucial role in the development of OSCC by modulating cell growth, migration, and metastasis.

**Supplementary Information:**

The online version contains supplementary material available at 10.1007/s12672-025-03648-z.

## Introduction

Oral squamous cell carcinoma (OSCC) is one of the deadliest head and neck malignancies worldwide. Squamous cell carcinoma exhibits strong local migration and invasion, which result in the movement of tumour cells from a primary site to the cervical lymph nodes [1, 2]. In consideration that metastasis of squamous cell carcinoma is a crucial cause of cancer death, uncovering potential regulatory mechanism in the pathophysiological progression of cancer metastasis and identification of effective biomarkers will hopefully contribute to the development of new strategies against the metastasis of OSCC.

It is well known that proliferation, apoptosis, cell cycle and migration play important roles in regulating many metastasis processes of OSCC [3–5]. The cell division cycle associated 8 (CDCA8) gene encodes the Borealin/Dasra B protein and is a component of the chromosome passenger complex (CPC). Existing evidence revealed that CDCA8 was highly expressed in the tumorigenesis including lung cancer and breast cancer [6, 7]. CDCA8 is essential for cellular proliferation and innate immune responses [8, 9]. Tumor metastasis-associated signaling pathways, such as apoptosis, cell cycle and mTOR, were enriched in the CDCA8 high expression phenotype according to gene set enrichment analysis from The Cancer Genome Atlas (TCGA) database, and abnormal CDCA8 expression is dramatically linked with the development of liver cancer. Besides, inhibition of CDCA8 was also shown to suppress the growth of hepatocellular carcinoma by repressing AKT/β–catenin signaling pathway [10, 11]. However, the function of CDCA8 during processes of OSCC and the exact molecular mechanisms remain unknown.

Cyclin-dependent kinase 1 (CDK1) and CDK2, as the essential cell cycle drivers, offer an ideal target for cell cycle modulation at the G2/M transition [12, 13]. The aberrant CDK1 expression will induce the dysfunction of cell function and cause different pathological conditions in organs, such as cell transformation and tumorigenesis [14, 15]. Decreased expression of CDK1 in colorectal cancer cells caused S phase cell cycle arrest, thereby suppressing subcutaneously transplanted tumor growth [16]. It was demonstrated that CDK1 served as the downstream molecule of CDCA8, which contribute to the development and progression of thyroid cancer [17]. Similarly, another study demonstrated that inhibition of CDK1 was as an effective therapeutic agent for pancreatic cancer [18]. Therefore, we hypothesize that CDCA8 in oral cancer cells may modulate cell proliferation, apoptosis, cell cycle and migration via CDK1, then have an impact on OSCC.

This study focuses on investigating the potential molecular mechanism of OSCC and exploring the possible therapeutic strategy. Firstly, we observed the changes of proliferation, apoptosis, cell cycle and migration in oral cancer cells after silencing CDCA8 using short hairpin RNA (shRNA). Then, the possible signaling pathways, which is activated by CDCA8, during the development of OSCC were further investigated by TCGA database, KEGG pathway enrichment analysis and the intervence of CDK1. At last, we verified the effect of depletion of CDCA8 on improving the progression of OSCC based on a mice model. The findings from the present study will prove useful in expanding our understanding of the molecular mechanism of oral squamous cell carcinoma.

## Materials and methods

### Chemicals and reagents

The apoptosis kit (88-8007) was from eBioscience (CA, USA). Antibodies against CDCA8 (ab67126), CCNA2 (ab32386), CCNB1 (ab32053), CCNB2 (ab250841), CDK1 (ab133327), CDK2 (ab32147), PLK1 (ab189139), E-cadherin (ab231303) and Vimentin (ab20346) were from Abcam (Cambridge, MA). Antibody against GAPDH (AP0063) was from Bioworld Technology (USA). CCK8 kit (96992) and antibody-propidiumIodide (PI) (P4170) were purchased from Sigma-Aldrich (St. Louis, USA).

### Clinical samples and immunohistochemistry

45 patients diagnosed with OSCC from in the Northern Jiangsu People’s Hospital Affiliated to Yangzhou University were recruited for this study from May 2020 to December 2022. Subsequently, we obtained 45 tumor tissues, 9 normal tongue tissues. All patients did not receive chemotherapy or radiotherapy pre-surgery. OSCC tissues were collected during surgery. All cases were staged according to the American Joint Committee on Cancer (AJCC) 7th edition cancer staging manual. Clinical information and pathological data were collected from patients. The study was approved by the Ethics Committee of Northern Jiangsu People’s Hospital Affiliated to Yangzhou University (2024ky036) and was conducted in accordance with the Declaration of Helsinki and relevant institutional regulations. All patients signed a written informed consent before participation in the study.

IHC assays were performed as previously described [19]. After antigen repair, tumor tissue sections were incubated overnight at 4 °C with CDCA8 antibody (ab67126, 1: 200 dilutions, Abcam, Cambridge, MA, USA). Cells were then detected using the ChemMate DAKO EnVision Assay Kit (DAKO, Copenhagen, Denmark). Finally, sections were restained with Mayer hematoxylin. Staining was assessed by three independent researchers.

CDCA8 staining was scored on a scale referenced to Hongxia Dan’s method [20]. Specifically: staining intensity: 0-no staining seen, 1-pale yellow, 2-medium yellow, 3-dark yellow, or 4-brown; and percentage of staining: 1 (≤ 10%), 2 (10–50%), 3 (50–80%), or 4 (≥ 80%). The product of the two scores was used as the final score (9 grades in total: 1, 2, 3, 4, 6, 8, 9, 12 and 16). Scores of 1, 2, 3, and 4 were considered CDCA8 low expression; scores of 6, 8, 9, 12, and 16 were considered CDCA8 high expression.

### In vitro experiments

**Fig. 1:**
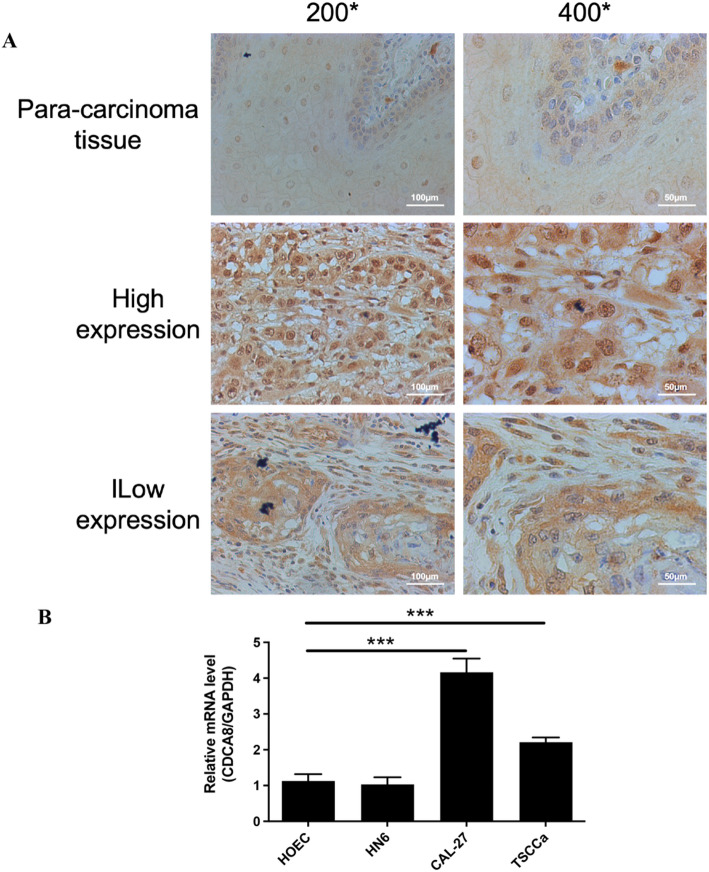
CDCA8 was highly expressed in oral cancer. **A** IHC analysis of CDCA8 protein expression in OSCC tissues (original magnification: 200× or 400×; Scale: 100 μm–50 μm). **B** RT-qPCR assay of CDCA8 mRNA relative expression in HOEC, HN6, CAL-27 and TSCCa cells. *P < 0.05; **P < 0.01; ***P < 0.001

Human oral cancer cell line HN6, CAL-27 and TSCCa cells were obtained from the Procell Life Science&Technology Co., Ltd. Human oral epithelial cells (HOECs) were obtained from the Chinese Academy of Sciences Cell Bank (China). All cells were cultured in DMEM (Gibco, USA) medium containing 10% fetal bovine serum (FBS) and 1% penicillin/streptomycin (Fig. 1).

**Fig. 2:**
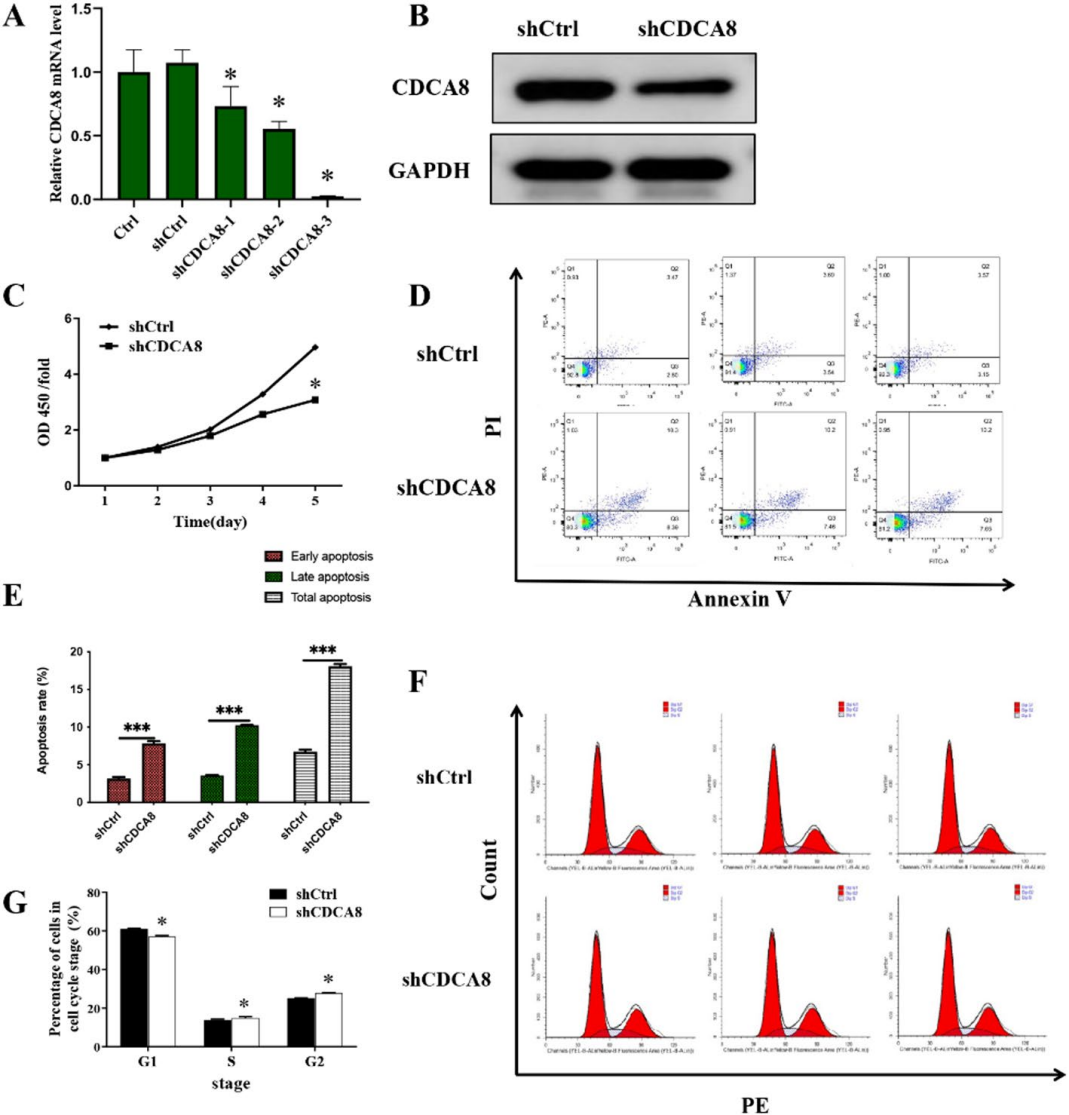
CDCA8 deficiency in CAL-27 cells suppressed proliferation and enhanced apoptosis. CAL-27 cells were transfected with shCDCA8-1, shCDCA8-2 and shCDCA8-3 for 72 h. After transfecting, CAL-27 cells were collected. **A** RT-qPCR assay of CDCA8 mRNA relative expression in CAL-27 cells. **B** Western Blotting analysis of CDCA8 protein expression in CAL-27 cells after transfecting with shCDCA8-3 for 72 h. **C** CCK-8 assay was to assess the time effect of silencing CDCA8 on CAL-27 cells proliferation. **D** Representative scatter plot of PI^+^Annexin V^+^ and PI^−^Annexin V^+^ staining CAL-27 cells by flow cytometry analysis. **E** The percentages of cell early, late and total apoptosis were analyzed according to the different positive expressions of PI and Annexin V in CAL-27 cells. **F**, **G** CAL-27 cells cycle at G1, S and G2 phases were tested by flow cytometry analysis. *P < 0.05; **P < 0.01; ***P < 0.001

To identify the role of CDCA8 in OSCC in vitro, CDCA8 expression in CAL-27 cells was inhibited using lentiviral transfection, then cell proliferation, apoptosis, cell cycle and migration were evaluated. Specifically, CAL-27 cells were infected with lentivirus containing CDCA8 shRNA (shCDCA8 group) or negative control (shCtrl group) target sequences, and the cells were then cultured at 37 ℃ for 72 h. The recombinant lentivirus plasmid targeting shCDCA8-1 (GTGGAAATACGAATCAAGCAA), shCDCA8-2 (AGATGAAATGATAGTGGAAGA), shCDCA8-3 (GCGGAGAGAGCCTGCGATTAT) and the control shRNA (ACGACGTCAGCTGGTGCATGT) were created. Real-time quantitative polymerase chain reaction (RT-qPCR) results showed that shCDCA8-3 had the strongest silencing efficiency (Fig. 2A). Similarly, as shown in Fig. 2B, shCDCA8-3 treatment markedly decreased CDCA8 expression at protein level. Thus, shCDCA8-3 treatment is an effective way to silence CDCA8 in CAL-27 cells in vitro experiments. The CDCA8 gene was used as a template, and the primer amplification sequence was designed to construct the lentivirus of LV-013 vector. All overexpression and knockdown CDCA8 lentiviruses were synthesized by GeneChem (Shanghai, China). Subsequently CAL-27 cells underwent lentiviral infection process. Briefly, CAL-27 cells were infected with lentivirus in the presence of 5 g/mL polyglutamine (Sigma-Aldrich, St. Louis, MO, USA) for 48 h and then selected with 2 g/AmL puromycin (Sigma-Aldrich, St. Louis, MO, USA) for 7 days. Cells resistant to puromycin were isolated for further studies. To determine the causal association between CDCA8 and CDK1 in oral squamous cell carcinoma, CDK1 inhibitor RO3306 (2 mM) pretreatment was used to inhibit CDK1 expression in CAL-27 cells.

### Animal treatment and in vivo imaging

Four-week-old BALB/c mice were purchased from the GemPharmatech Co., Ltd. CAL-27 cells (1 × 10^7^) were subcutaneously inoculated into mice (shCtrl group, *n* = 5) to establish a model of OSCC. To investigate whether CDCA8 deficiency acts a protective role in the progression of OSCC, mice were subcutaneously injected with shCDCA8 (shCDCA8 group, *n* = 5, once a day) on day 10 after CAL-27 cells administration. Then, tumor tissues were obtained, as well as the sizes and morphological alterations of tumor tissues were evaluated on day 23 after CAL-27 cells injection. The IVIS Spectrum animal imaging system (LB983, Berthold Technologies) was used to evaluate the tumor growth and whole metastasis conditions with 100 µl XenoLight D-luciferin Potassium Salt (15 mg/ml, Perkin Elmer) per mouse on day 23. All procedures on mice followed the guidelines for humane treatment by Northern Jiangsu People’s Hospital Affiliated to Yangzhou University (202402881). The maximum permissible tumor volume for the mouse subcutaneous tumor model was set at 2000 mm^3^ or a tumor diameter of no more than 20 mm. the maximum permissible tumor volume or tumor diameter was not exceeded at any time during the animal experiments. This study was conducted in accordance with the ARRIVE (Animal Research: Reporting of In Vivo Experiments) guidelines for reporting animal studies. The mice were humanely euthanized by gradually increasing the CO₂ concentration in a sealed chamber until asphyxiation occurred.

### CCK-8 assay

To evaluate the change of proliferation in CAL-27 cells after shCDCA8 treatment, CCK-8 assay was performed. After infecting lentivirus with CAL-27 cells for various days (1, 2, 3, 4 and 5 days), 10 µl CCK-8 solution was directly added to culture medium of CAL-27 cells, then cells were incubated for 1 h at 37 ℃. The absorbance was measured at 450 nm and cell proliferation extent was calculated.

### Flow cytometry (FCM)

To analyze the percentage of CAL-27 cells apoptosis, CAL-27 cells were stained with phycoerythrin (PE)-PI and fluorescein isothiocyanate (FITC)-Annexin V after blocking nonspecific binding. To analyze the cell cycle, CAL-27 cells were stained with PI/RNase solution. Then, CAL-27 cells apoptosis and cycle were evaluated by FCM. PI^−^Annexin V^+^ and PI^+^Annexin V^+^ cells were considered as the early cell apoptosis and late cell apoptosis, respectively. The data were analyzed by *CytExpert 2.0* software.

### Transwell and wound healing assays

Transwell and wound healing assays were used to detect the rate of migration in CAL-27 cells. As for transwell assay, CAL-27 cells were cultured in the upper chamber of transwell system, then lentivirus infection was conducted. After 6 h, the non-migrating cells were fully removed, and the cells in the bottoms of the inserts were then fixed and stained with 0.1% crystal violet staining solution. The numbers of migrating cells were measured under a light microscope (Olympus, Tokyo, Japan). As for wound healing assay, pipette tip was used to scratch CAL-27 cells and create a wound. Next, the migration distance was calculated at 6 h after scratch administration.

### Western blotting

The total proteins of CAL-27 cells were extracted using RIPA lysis buffer, then concentration of proteins was measured and adjusted to the equal amounts. After separating by SDS–PAGE, proteins were transferred to polyvinylidene fluoride membranes. After blocking nonspecific binding, membranes were incubated with anti-CDCA8 (1:1000), anti-CCNA2 (1:1000), anti-CCNB1 (1:1000), anti-CCNB2 (1:1000), anti-CDK1 (1:1000), anti-CDK2 (1:1000), anti-PLK1, anti-E-cadherin (1:2000), anti-Vimentin (1:2000) and anti-GAPDH (1:2000) antibodies at 4 °C overnight. On the following day, membranes were incubated with corresponding second antibodies, and the signals were visualized using an enhanced ECL detection kit.

### RT-qPCR

Total RNA was extracted using TRIzol reagent, and cDNA was reverse-transcribed using a Promega M-MLV kit. RT-qPCR was performed with Light Cycler 480 SYBR Green I Master Mix. The PCR amplification reactions were conducted, then relative mRNA expressions were analyzed using the standard 2^−ΔΔCt^ method. The primer sequences used in this study are as follows: GAPDH, Forward sequence: 5’-TGACTTCAACAGCGACACCCA-3’; Reverse sequence: 5′- CACCCTGTTGCTGTAGCCAAA-3′; CDCA8, Forward sequence: 5′-GCAGGAGAGCGGATTTACAAC-3′; Reverse sequence: 5′-CTGGGCAATACTGTGCCTCTG-3′; CDK1, Forward sequence: 5′-CCTATGGAGTTGTGTATAAGGGT-3′; Reverse sequence: 5′-AGCACATCCTGAAGACTGACT-3′.

### Histological evaluation

The tumor tissues were preserved in 4% paraformaldehyde and prepared as paraffin-embedded sections. HE staining and immunohistochemical (IHC) were performed to evaluate the morphological alterations and CDCA8 or ki67 positive expression, respectively. The sections were observed by a light microscopy (Olympus, Tokyo, Japan) and representative images were acquired from tumor tissues in each group.

### Data collection

The head and neck squamous carcinoma transcriptome data were obtained from the TCGA dataset (TCGA-HNSC, https://portal.gdc.cancer.gov/) (Experimental Strategy: RNA-Seq; Data Category: Transcriptome Profiling; Data Type: Gene Expression Quantification; Access: open). A total of 521 head and neck squamous carcinoma tumor samples were obtained (Supplementary material 1). The median value of CDCA8 expression level was used to divide the cohort of TCGA database into high and low expression groups, and log2|FC|>2, *P* < 0.05 was used as the filtering criterion to do the differential analysis between the two groups. The differential genes obtained were subjected to KEGG enrichment analysis using the R clusterProfiler package, in order to explore the pathways involved in CDCA8.

### Statistical analysis

The data are presented as the *mean* ± *SD*. The experimental data for multiple group comparisons were processed by one-way ANOVA analysis and analyzed using *SPSS 23.0* software. Mann-Whitney U analysis was used to assess the relationship between the expression of CDCA8 gene expression in expressing patients features. Spearman correlation analysis was used to assess the correlation of CDCA8 expression with patient age and tumor stage. Visualization and Integrated Discovery was used to perform KEGG pathway enrichment analysis. Data are expressed as the mean ± standard deviation (SD) of three replicate wells from in vitro experiments and five mice per group, representing three biological replicates. *P* < 0.05 was considered significant.

## Results

### CDCA8 was highly expressed in oral cancer

To investigate the expression of CDCA8 in oral cancer, we recruited oral cancer patients to collect tumor tissues and para-carcinoma tissues. The IHC staining results of the tissues are displayed in Fig. 1A and found that the CDCA8 protein levels were upregulated in oral cancer tissues compared to the adjacent tissues (Table 1). According to the Mann-Whitney U analysis, the CDCA8 gene expression was significantly different in age and pathologic grade (*P* = 0.049 and 0.022, Table 2). Further, according to Spearman rank correlation analysis, it was shown that the expression of CDCA8 gene was positively correlated with age and pathological grading (Table 3). That is, the expression of CDCA8 gene increased as the age of patients increased, and the malignancy of tumors deepened.

**Table 1:** Expression patterns in oral squamous cell carcinoma tissues and para-carcinoma tissues revealed in immunohistochemistry analysis

CDCA8 expression	Tumor tissue		Para-carcinoma tissue		p value
	Cases	Percentage	Cases	Percentage	
Low	20	44.4%	9	100%	0.000***
High	25	55.6%	0	0%	

**Table 2:** Relationship between CDCA8 expression and tumor characteristics in patients with oral squamous cell carcinoma

Features	No. of patients	CDCA8 expression		*p* value
		low	high	
All patients	45	20	25	
Age (years)				0.049*
< 58	33	14	19	
≥ 58	12	6	6	
Grade				0.228
Low	38	18	20	
High	7	2	5	
TNM				0.085
1	7	4	3	
2	24	11	13	
3	8	3	5	
4	6	2	4	
Stage				0.022*
1	1	0	1	
2	14	10	4	
3	17	3	14	
4	13	7	6	

**Table 3:** Relationship between CDCA8 expression and tumor characteristics in patients with oral squamous cell carcinoma

		CDCA8
Age	Spearman’s correlation	− 0.300*
	Significance (two-tailed)	0.048
	N	45
Stage	Spearman’s correlation	− 0.349*
	Significance (two-tailed)	0.020*
	N	45

### CDCA8-deficient oral cancer cells reduced proliferation and increased apoptosis

The expression of CDCA8 was also analyzed in OSCC cell lines. As shown in Fig. 1B, CDCA8 expression was significantly upregulated in OSCC cell lines CAL-27 and TSCCa compared to human oral epithelial cells (HOECs), while its expression in HN6 cells showed minimal change. Notably, CAL-27 exhibited the most pronounced increase in CDCA8 expression and was therefore selected for subsequent experimental validation. Then, oral cancer cells proliferation and apoptosis were investigated after silencing CDCA8 in vitro to clarify the regulatory mechanism of CDCA8 in the course of OSCC (Fig. 2A-B). CCK-8 results showed that shCDCA8 treatment decreased the cell growth as the increasing treatment days, most prominent on day 5 after shCDCA8 administration (Fig. 2C). As presented in Fig. 2D-E, FCM results demonstrated that the percentage of positive expression of Annexin V in CAL-27 cells in shCDCA8 group was dramatically elevated compared to that in shCtrl group. In particular, the percentage of cell late apoptosis was significantly higher after shCDCA8 administration, as well as shCDCA8 did not induce the change of the percentage of cell early apoptosis. Furthermore, CDCA8-deficient CAL-27 cells exhibited decreased cell percentage at the G1 phase and increased cell percentage at the G2 phase (Fig. 2G-H). Together, these data show that CDCA8 deficiency reduces proliferation, increases apoptosis, and causes cell cycle arrest in oral cancer cells.

### CDCA8-deficient oral cancer cells reduced the capacity of migration

**Fig. 3:**
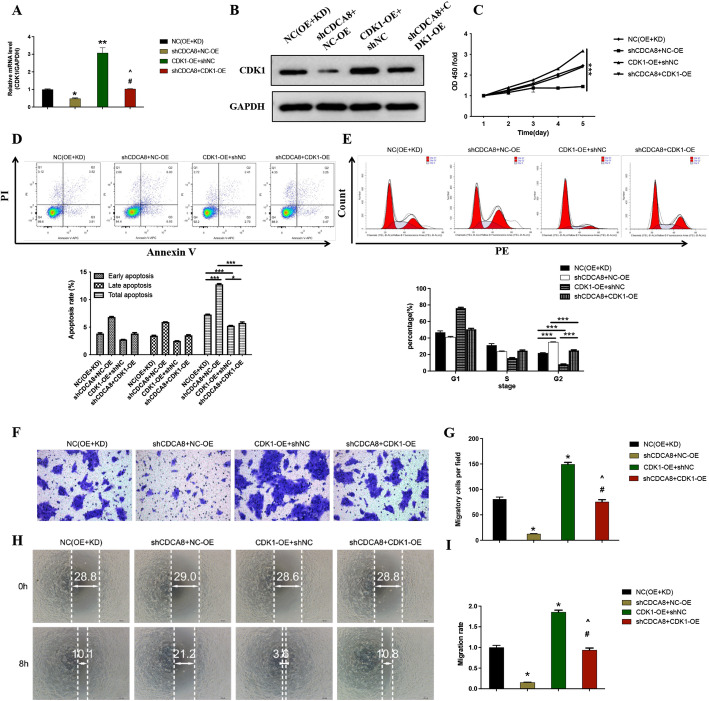
CDCA8 deficiency in CAL-27 cells suppressed migration activity. The wound-healing assay (**A**, **B**) and transwell assay (**C**, **D**) were performed to assess the effect of CDCA8 deficiency on the migration rate of CAL-27 cells. ***P < 0.001

To gain information about the change of migration in CDCA8-deficient oral cancer cells, we performed wound healing assay and transwell assay. As shown in Fig. 3, when in contrast to the control CAL-27 cells, the migration activity was significantly inhibited by shCDCA8. Therefore, our findings suggest that CDCA8 deficiency may act a protective role in the development of OSCC.

### The correlation of CDCA8 and CDK1 during the development of OSCC

**Fig. 4:**
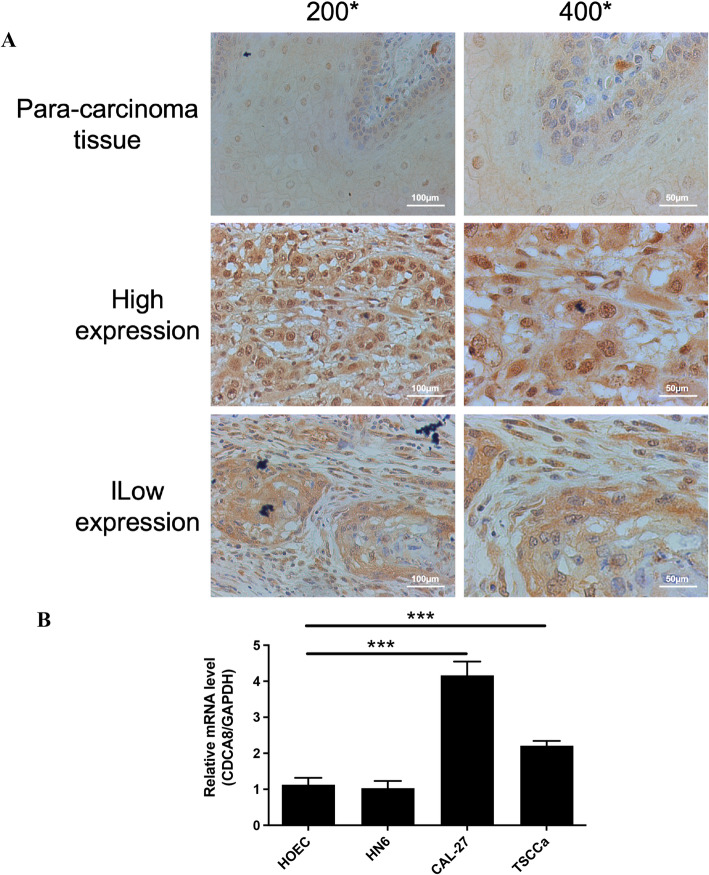
CDCA8-mediated squamous cell carcinoma of oral required CDK1 activation. **A** Data were obtained from TCGA data, then the correlation of CDCA8 and related signaling pathways was analyzed and performed as a bubble diagram. **B** The key role of CDCA8 in cell cycle-related pathway. **C** Western blotting analysis of CDCA8, CCNA2, CCNB1, CCNB2, CDK1, CDK2 and PLK1 protein levels in CAL-27 cells

Next, we aimed to investigate the potential downstream cooperator of CDCA8, we found 28 signaling pathways were closely associated with CDCA8 expression through TCGA database. Among then, a strong positive correlation of CDCA8 and cell cycle pathway was observed (Fig. 4A). These findings prompted us to further focus on cascade correlation between CDCA8 and cell cycle pathway.

KEGG analysis showed a possible involvement of CDCA8 in the regulation of CDK1 in CAL-27 cells (Fig. 4B). To further verify this hypothesis, we detected the protein expressions of CCNA2, CCNB1, CCNB2, CDK1, CDK2 and PLK1 in CDCA8-deficient CAL-27 cells. As expected, CDCA8 protein expression was dramatically inhibited by shCDCA8 treatment. No obvious changes of CCNB1, CCNB2 and PLK1 protein expressions were observed between shCTRL group and shCDCA8 group. However, silencing CDCA8 downregulated levels of CCNA2, CDK1 and CDK2 (Fig. 4C). Therefore, the data indicate an involvement of CDCA8 in CDK1, which regulate cell cycle signaling during OSCC.

### Suppressing CDK1 reduced CDCA8 overexpression-induced proliferation and migration in CAL-27 cells

**Fig. 5:**
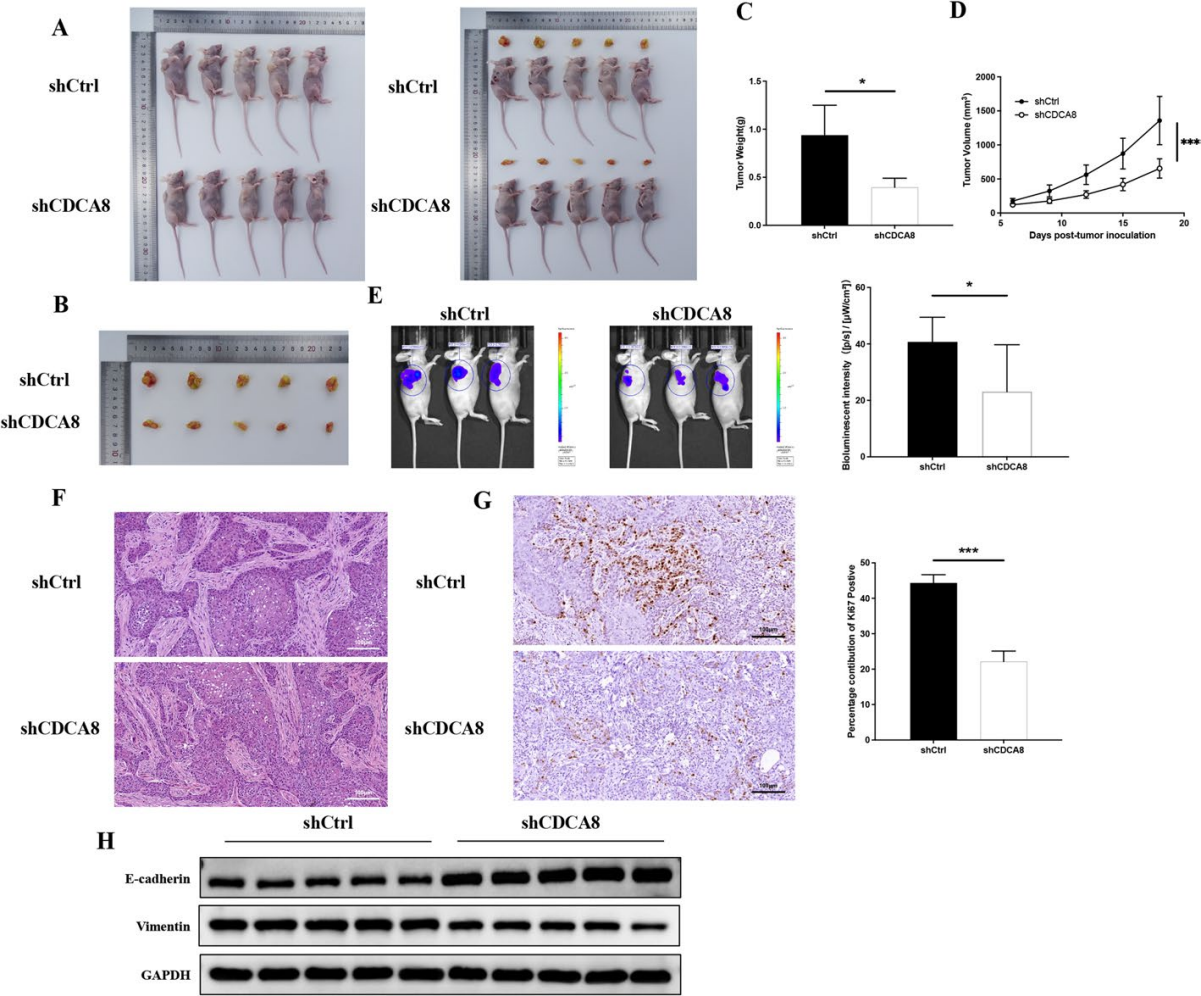
Inhibition of CDK1 reversed CDCA8 overexpression-induced proliferation and migration in CAL-27 cells. CDK1 inhibitor was used to inhibit CDK1 expression in CAL-27 cells based on CDCA8 overexpression treatment. **A** Western blotting analysis of CDK1 protein expression in CAL-27 cells. **B** CCK-8 assay was to assess the effect of silencing CDK1 on CAL-27 cells proliferation after CDCA8 overexpression treatment. **C** The wound-healing assay was performed to assess the effect of silencing CDK1 on the migration rate of CAL-27 cells. ***P < 0.001 vs. NC group; ^###^*P* < 0.001 vs. CDCA8 group

CDCA8 overexpression and CDK1 inhibitor treatments were used to test the causal association between CDCA8 and CDK1 in oral squamous cell carcinoma. Results showed that CDK1 inhibitor reduced CDCA8 overexpression elevated CDK1 expression (Fig. 5A). Meanwhile, the elevated capacity of proliferation and migration induced by CDCA8 overexpression was markedly inhibited by CDK1 inhibitor (Fig. 5B-C). These findings demonstrate that CDCA8/CDK1 pathway regulates proliferation and migration in oral cancer cells.

### Overexpression of CDK1 alleviated the inhibitory effects of CDCA8 knockdown in CAL-27 cells

**Fig. 6:**
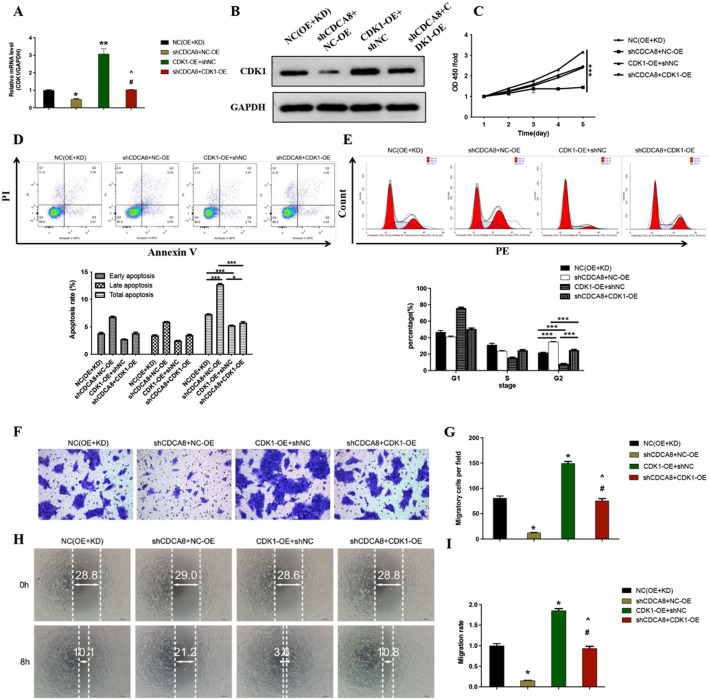
Overexpression of CDK1 alleviated the inhibitory effects of CDCA8 knockdown in CAL-27 cells. **A** RT-qPCR assay of CDK1 mRNA relative expression in CAL-27 cells. **B** Western Blotting analysis of CDK1 protein expression in CAL-27 cells. **C** CCK-8 assay was to assess CAL-27 cells proliferation. **D** Representative scatter plot of PI + Annexin V + and PI-Annexin V + staining CAL-27 cells by flow cytometry analysis. **E** CAL-27 cells cycle at G1, S and G2 phases were tested by flow cytometry analysis. The transwell assay (**F**, **G**) and wound-healing assay (**H**, **I**) were performed to assess the migration rate of CAL-27 cells. *P < 0.05 vs. NC (OE + KD) group; #*P* < 0.05 vs. shCDCA8 + NC-OE group; ^*P* < 0.05 vs. CDK1-OE + shNC group

As expected, overexpression of CDK1 effectively upregulated mRNA and protein levels of CDK1 (Fig. 6A-B). Furthermore, the results of CCK-8 showed that compared with the normal cells, the cell proliferation rate and the capacity of migration in the CDK1-OE + shNC group were the strongest, followed by the shCDCA8 + CDK1-OE group, suggesting that overexpression of CDK1 reversed shCDCA8-induced inhibitory effects of cell proliferation rate and the capacity of migration in CAL-27 cells (Fig. 6C, F-I). In contrast, overexpression of CDK1 has a certain inhibitory effect on shCDCA8-enhanced apoptosis in CAL-27 cells (Fig. 6D). Regarding cell cycle, CDK1-OE + shNC group had the most significant inhibitory effect on cell percentage at the G2 phase, while shCDCA8 + CDK1-OE group reversed this effect (Fig. 6E). Collectively, CDCA8/CDK1 axis exert a role in promoting the development and progression of OSCC.

### CDCA8 depletion effectively alleviated OSCC in vivo

**Fig. 7:**
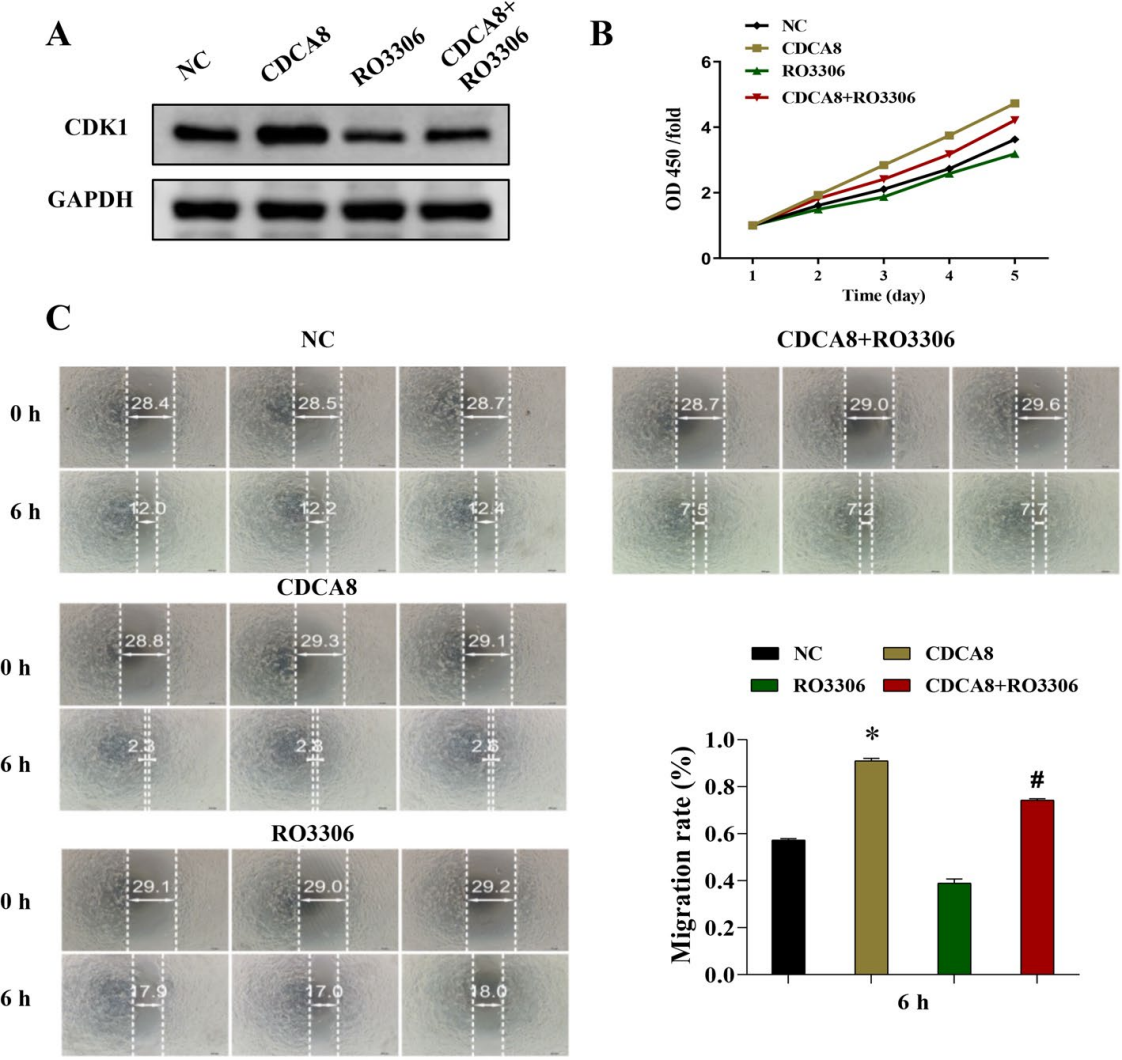
CDCA8 depletion improved squamous cell carcinoma of oral in mice. (**A**, **B** The size of tumors in mice. **C** The metastasis of oral squamous cell carcinoma was observed by in vivo imaging system. **D** Histological alterations of tumors were observed using HE staining (original magnification: 200×; Scale: 100 μm). **E** Immunohistochemistry of ki67-positive expression in tumors of mice (Original magnification: 400×; Scale: 100 μm). **F** Evaluation of epithelial-mesenchymal transition level (E-cadherin and Vimentin expressions) by Western Blotting

At last, we aimed to determine whether CDCA8 depletion inhibits OSCC based a mice model. Tumor volume and mass were greatly suppressed after treatment with shCDCA8 (Fig. 7A-D). As shown in Fig. 7E, results in vivo imaging system mice showed that in shCtrl group presented obvious metastasis of tongue OSCC, and shCDCA8 administration efficiently suppressed cancer metastasis on day 23 compared to the control mice. HE staining showed that mice exhibited a lower number of cancer foci post shCDCA8 injection (Fig. 7F). The positive expression of ki67 in tumor tissues, which has been reported to be associated with cell proliferation, was determined by IHC. As presented in Fig. 7G-H, compared with those control mice, CDCA8-depleted mice formed a decreased in ki67-positive tumor cells and epithelial-mesenchymal transition level, characterized by increased E-cadherin and decreased Vimentin. As above, our findings indicate that CDCA8 depletion effectively alleviates OSCC via inhibiting tumor cells proliferation in mice.

## Discussion

In the present study, we illustrated that CDCA8 deficiency played a protective role in OSCC in vitro via inhibiting proliferation and migration and promoting apoptosis in CAL-27 cells. Additional KEGG pathway enrichment analysis demonstrated that CDK1 was strongly associated with CDCA8 during OSCC pathogenesis. Finally, we verified depletion of CDCA8 or CDK1 effectively alleviated the metastasis of OSCC. Our findings from the current study provide the possibility that inactivating CDCA8/CDK1 pathway serve as a promising strategy to counteract and treat OSCC.

CDCA8 act as a key molecular regulator of proliferation, cell cycle, metabolism and metastasis, which have been implied in the involvement of tumor development [11, 21, 22]. The effect of CDCA8 on cell cycle and metastasis has been studies in several tumor cells, such as human glioma cell and osteosarcoma cells [23, 24]. Bi et al. reported that bladder cancer tissues from patients displayed an aberrant overexpression of CDCA8, which was associated with poor clinicopathological features of bladder cancer, thus CDCA8 might serve as a biomarker to evaluate the severity of bladder cancer in patients and act as a novel target for tumor therapy [25]. Similarly, one study in vitro also found that CDCA8 was markedly overexpressed in prostate cells, and inhibition of CDCA8 obviously reduced proliferation and migration in prostate cells [26]. However, there is insufficient evidence on the association between CDCA8 and OSCC, as well as the detailed downstream mechanism of CDCA8 is not clear. The tissue homeostasis is maintained through a balance of proliferation and apoptosis signals, and severity of tumor metastasis depends on the capacities of migration and invasion of tumor cells [27–30]. Consistent with these findings, our findings demonstrated that depletion of CDCA8 displayed an obvious anti-tumor effect in mice, characterized by small size of tumor tissues, decreased cancer metastasis and proliferation of tumor cells. In vitro experiments, CDCA8-deficient oral cancer cells exhibited a decrease in proliferation and migration and an increase in apoptosis, as well as led to cell cycle arrest at G2 stage. Our findings imply that CDCA8 is a potent inducer and prognostic marker in the progression of OSCC, and inhibition of CDCA8 may serve a therapeutic target for OSCC.

To gain an insight into the downstream signaling pathway of CDCA8 in OSCC, data were obtained from TCGA database and KEGG analysis was performed. We found that there was a strong correlation between CDCA8 and cell cycle signaling pathway, as well as CDK1 was the key regulatory molecular. CDK1 has been highlighted as an important downstream effector of CDCA8 and transcriptional regulator associated with the tumor metastasis. Lu et al. reported that activation of CDK1 activity regulated Bcl-2-mediated mitotic arrest and apoptosis [31]. In our results, according to the TCGA database and KEGG analysis, CDK1 is highly expressed in squamous cell carcinoma and is closely associated with CDCA8, and CDK1 expression was greatly inhibited by shCDCA8. CDCA8 may activate CDK1-mediated the regulation of proliferation, migration, invasion and apoptosis. To verify this finding, CDK1 inhibitor was used to treat CAL-27 cells, and we confirmed that CDK1 was activated by CDCA8, thereby promoting OSCC development and progression.

Our study still has some limitations. The potential molecular mechanism between CDCA8 and CDK1 of cell cycle pathway still need to be further investigated. In addition, future study is warranted to elucidate whether CDK1 inhibitor could alleviate oral cavity carcinomas in vivo.

## Conclusion

Taken together, depletion of CDCA8 in oral cancer cells alleviates OSCC by modulating cell proliferation, apoptosis, cell cycle and migration. Among them, cell cycle, which was regulated by CDK1, play the most prominent effect on the progression of OSCC.

## Supplementary Information

Below is the link to the electronic supplementary material.

Supplementary Material 1.

## Data Availability

The datasets analyzed during the current study are openly available from The Cancer Genome Atlas (TCGA) database, (TCGA-HNSC, URL: https://portal.gdc.cancer.gov/). Additionally, the original sequencing data files from TCGA are available for download in the Supplementary Materials.
